# Muscle synergies in joystick manipulation

**DOI:** 10.3389/fphys.2023.1282295

**Published:** 2023-10-13

**Authors:** Liming Cai, Shuhao Yan, Chuanyun Ouyang, Tianxiang Zhang, Jun Zhu, Li Chen, Xin Ma, Hui Liu

**Affiliations:** ^1^ Academy for Engineering and Technology, Fudan University, Shanghai, China; ^2^ Suzhou Institute of Biomedical Engineering and Technology, Chinese Academy of Sciences, Suzhou, China; ^3^ School of Biomedical Engineering (Suzhou), Division of Life Sciences and Medicine University of Science and Technology of China, Suzhou, China; ^4^ Department of Orthopedics, Huashan Hospital, Fudan University, Shanghai, China; ^5^ Cognitive Systems Lab, University of Bremen, Bremen, Germany

**Keywords:** muscle synergy extraction, muscle synergy similarity, joystick manipulation, maneuvering, electromyography, EMG

## Abstract

Extracting muscle synergies from surface electromyographic signals (sEMGs) during exercises has been widely applied to evaluate motor control strategies. This study explores the relationship between upper-limb muscle synergies and the performance of joystick manipulation tasks. Seventy-seven subjects, divided into three classes according to their maneuvering experience, were recruited to perform the left and right reciprocation of the joystick. Based on the motion encoder data, their manipulation performance was evaluated by the mean error, standard deviation, and extreme range of position of the joystick. Meanwhile, sEMG and acceleration signals from the upper limbs corresponding to the entire trial were collected. Muscle synergies were extracted from each subject’s sEMG data by non-negative matrix factorization (NMF), based on which the synergy coordination index (SCI), which indicates the size of the synergy space and the variability of the center of activity (CoA), evaluated the temporal activation variability. The synergy pattern space and CoA of all participants were calculated within each class to analyze the correlation between the variability of muscle synergies and the manipulation performance metrics. The correlation level of each class was further compared. The experimental results evidenced a positive correlation between manipulation performance and maneuvering experience. Similar muscle synergy patterns were reflected between the three classes and the structure of the muscle synergies showed stability. In the class of rich maneuvering experience, the correlation between manipulation performance metrics and muscle synergy is more significant than in the classes of trainees and newbies, suggesting that long-term training and practicing can improve manipulation performance, stability of synergy compositions, and temporal activation variability but not alter the structure of muscle synergies determined by a specific task. Our approaches and findings could be applied to 1) reduce manipulation errors, 2) assist maneuvering training and evaluation to enhance transportation safety, and 3) design technical support for sports.

## 1 Background

Fine manipulation refers to the ability to perform precise actions or delicate tasks using hands or tools, presented as coordination and precision of movements (Tang et al., 2017; Tang et al., 2021). In the training and selection of pilots, evaluating the control ability of the joystick is critical, as the joystick is one of the primary control devices in an aircraft and common interactive tools applied with muscle synergy. In (Bezerra et al., 2019), the mean and peak electromyographic activation values during joystick operation have been studied for favorable pilot load conditions. Previous research also revealed that muscle coordination changes during motor adaptation to the viscous force field generated by haptic interface (Fabio et al., 2016) or visuomotor rotation (Gentner et al., 2013).

Joystick manipulation is a multi-joint coordinated motion for which numerous control modes exist in the motion control of the musculoskeletal system. Movements are inherently variable and can be as consistent as possible by repetition of professional athletes. Various studies have evidenced that the central nervous system (CNS) can generate motor commands through a series of synergistic combinations of muscles (d’Avella et al., 2003; Bizzi et al., 2008; Bizzi and Cheung, 2013), known as muscle synergy. Muscle synergy has recently been considered a common method for studying complex motor control patterns, applied to research fields of nerve damage effects (Roh et al., 2013; Tang et al., 2017), human posture control (Torres-Oviedo and Ting, 2007; Robert and Latash, 2008; Asaka et al., 2011), and locomotion of robot-assisted technology (Pham et al., 2011; Salman et al., 2010; Wang et al., 2021a; Wang et al., 2021b; Scano et al., 2019). Muscle contractions during exercise can be divided into isometric and isotonic. In isometric tasks, the module structures of muscle synergies are robust to changes in speed, and the neural commands to muscle synergies change in response to speed changes (Kojima et al., 2017). The motor control strategy can be modified depending on the accuracy requirement of the isometric reach task (Ettema et al., 2005a; Sano et al., 2020). In joystick isometric dual degree-of-freedom torque tasks, the torque history affects coordination patterns, and the motor system controls different orthogonal combinations of torque oscillations and constant torques employing a single oscillating muscle synergy (Ettema et al., 2005b). In joystick isometric force feedback tasks (Camardella et al., 2021), divided muscle synergies into groups with specific motor strategies and the same global groups for all subjects, confirming that muscle sets can be reduced to achieve comparable hand force estimation performances. For isotonic contraction tasks, the literature shows that the CNS can perceive the relationship between stiffness level and the size of the endpoint deviation (Gribble et al., 2003; Osu et al., 2004) and alter the mechanical impedance of the limb through the simultaneous activation of antagonist muscle groups (Feldman and Levin, 1995; Burdet et al., 2001), revealing the critical role of co-contraction in arm movement performance.

In addition to co-contraction indices, inter- and intra-subject similarities are hotspots in motor coordination (Alnajjar and Shimoda, 2017; Barnamehei et al., 2018b; Esmaeili and Maleki, 2020). Studies have indicated that muscle synergies are a valuable tool for quantifying variability at the muscle level and reveal that intra-subject variability is lower than inter-subject variability in synergy modules and related temporal coefficients, and both intra-subject and inter-subject similarities are higher than random synergy matching, confirming shared underlying control structures (Zhao et al., 2021). During specific movements, two indices of muscle cooperative stability and cooperative space are introduced for the CNS, characterized by the best recruitment mechanism in the process of balance behavior proficiency (Alnajjar and Shimoda, 2017). Taking kicking as an example, elite players have a high similarity in the stability of muscle synergies (Barnamehei et al., 2018b). In a controlled experiment with overhead shots, individual muscle patterns ranged from moderate to highly similar between elite and non-elite groups (Barnamehei et al., 2018a). These results suggest that the CNS can create optimal sets of efficient behaviors by optimizing the size of the synergy space in the appropriate region by interacting with the environment (Alnajjar et al., 2013). However, the group comparison did not correlate muscle synergy results with the performance of each exercise.

Training is hypothesized to change manipulation performance and muscle synergies, and there is a specific correlation between them. Subjects with different maneuvering experiences are selected to perform the rocker manipulation task. This study explores an objective and effective method to explore the relationship between muscle synergy and manipulation performance, providing theoretical support for sports science and scientific training methods. To the best of our knowledge, this article is the first to study the relationship between muscle synergy and joystick manipulation position accuracy. This fine activity is practical and essential for sports and transportation, rather than the broader sense movements of the limb or joint, often studied in related fields.

## 2 Data preparation: subjects, hardware, and setup

### 2.1 Subjects, classes, and ethics

Seventy-seven healthy right-handed male subjects, detailed in Table 1, aged 17–55 (25.18 ± 13.73) years, volunteered to participate in this study and were divided into three classes according to their maneuvering experience.

**TABLE 1:** Grouping and information of the 77 subjects.

Class	Number	Age	Maneuvering experience (flight time)
Experts	19	40–55	≥3,000 h
Trainees	30	18–19	7–8 h
Newbies	28	17–19	0
All	77	17–55	—

• Class Experts consists of professional pilots with over 1,000 h of flight time;
• Class Trainees is formed by short-haul experienced pilots who have completed 7–8 h of flight time in the company of professionals;
• Class Newbies contains high school students without experience in flying missions or joystick operations.

All subjects were informed of the experiment procedure approved by the Ethics Review Committee of Fudan University (No. FE22064R).

### 2.2 Experimental apparatus

**FIGURE 1:**
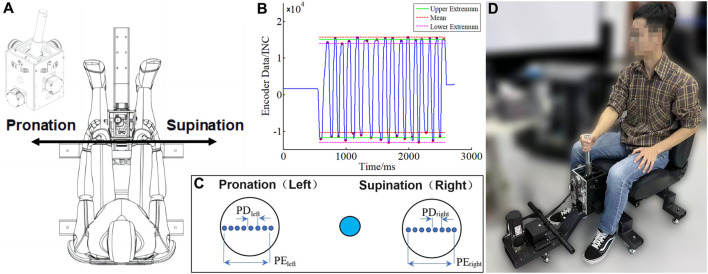
Self-developed experimental device and software interface. **(A)** Top view of the device for experiments; **(B)** An exemplar result record. PD: position deviation; PE: extreme range of the position. **(C)** A screenshot of the software interface; **(D)** Schematic experimental scene.

Figure 1A exhibits our self-developed experimental apparatus achieving rocker *X*/*Y* operation in two directions, for which the motor encoder recorded the position of the joystick in real-time. Users operate the experimental apparatus, interacting with an upper computer interface. Figures 1B,C exemplify one record of a trail.

The angle of the joystick ranges from ±15°, normalizing the angle to the spacing of the two black circles on the screen. The precision of the joystick is determined by the clearance of the planetary reducer, which has a backlash of two arc minutes, that is, 0.03 mm on the screen. The sensitivity of the device is determined by the resolution of the motor encoder, 0.005 mm. The self-implemented prototype acquired position data with a position sampling frequency of 200 Hz.

The coordination of the shoulder, elbow, wrist, and fingers enables the manipulation of the joystick. The reciprocating motion in this study is designed under a constant load condition (see Figure 1B). The internal and external rotation of the shoulder and the pronation/supination of the forearm were performed during the experiment, while the elbow joint was retracted in the coronal plane and flexed/extended in the sagittal plane. The range of motion of the forearm was more evident than that of the elbow and shoulder joints.

Figure 1D illustrates the schematic experimental scene. All subjects carried out experiments in their own habitual postures that remained consistent and could have a slight forward lean. The base of the experimental apparatus was fixed, and the seat backrest was lowered backward to maintain enough space for the human body. Therefore, no contact between the electrodes and the backrest was observed or detected in the experiment.

### 2.3 Acquisition details

For convenience in the discussion, the entire experimental procedure was described in terms of *supination* and *pronation*. During the experiment, subjects were asked to sit upright on the chair. The joystick moved back and forth along the *X*-axis, as depicted in Figure 1A. The blue ball (10 mm) and the black circle (20 mm) were initialized in the center and on the left/right sides, respectively (see Figure 1C); the joystick moved right along the +*X*-axis, while the blue ball synchronized with the right circle and *vice versa*.

Subjects were asked to maintain a certain distance between their elbows and thighs, thus exerting force with the upper limbs. They were required to place the blue ball precisely in the circle center as quickly as possible while maintaining accuracy. The blue ball reciprocated between the black circles, providing instant feedback on user operations while prioritizing accuracy. All participants performed 16 repetitions in each test to ensure that at least 14 were completed. The manipulation procedures and the corresponding sEMG data were recorded simultaneously.

Operating the rocker arm requires rotation of the forearm and the shoulder. The internal rotation of the shoulder mainly involves the anterior deltoid (DA), the pectoralis major (PM), the latissimus dorsi, and the teres minor (TM), while the external rotation involves the infraspinatus (INF), the teres minor (TM), and the posterior deltoid (DP). The energy of inner rotation is much higher than that of outer rotation (Ijiri et al., 2020). The biceps brachii (short/long head) can be used as a supinator muscle, where the biceps play a role in the activity of medium or high-power supination. The triceps brachii (long/lateral head) can be activated by the same length and neutralize the tendency of the biceps to bend the elbow (Soubeyrand et al., 2017). The pronator muscle consists of the pronator muscle and the pronator teres muscle. The brachioradialis (BRAD) is used primarily for elbow flexion but can also be used for pronation.

**FIGURE 2:**
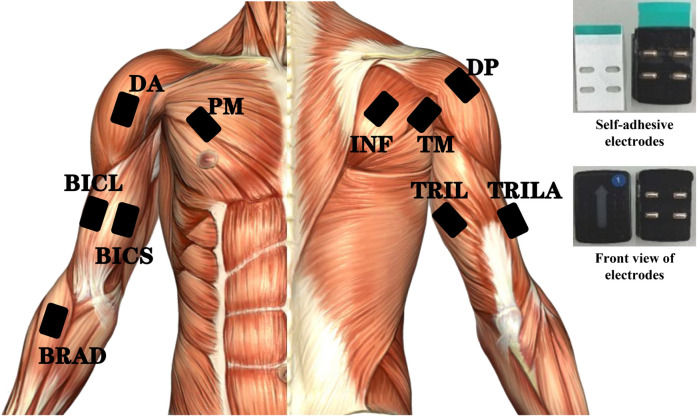
Placement and sensing muscles of sEMG sensors. Top right: self-adhesive electrodes. BRAD: brachioradialis; BICS: the short head of biceps; BICL: the long head of biceps; PM: pectoralis major; DA: anterior deltoid; TRIL: the long head of triceps; TRILA: the lateral head of triceps; INF: infraspinatus; TM: teres minor; DP: posterior deltoid.

As exhibited in Figure 2, a DELSYS Trigno system was used to collect sEMG and acceleration data from 10 muscles of the upper arm and shoulder, including BRAD, short head of the biceps (BICS), long head of the biceps (BICL), PM, DA, long head of the triceps (TRIL), lateral head of the triceps (TRILA), INF, TM, and DP. The placement of the sEMG electrodes follows the guidelines for noninvasive electromyographic assessment of muscles, as shown in Figure 2, at a sampling rate of 1,249 Hz, while the acceleration was sampled at 149 Hz. The acquired signals were processed by *MATLAB* (R2017b, MathWorks Natick, United States).

## 3 Methodology

### 3.1 Processing of motion encoder data

The position data were derived from the motor encoder (see Figure 1C) and processed in the following steps to provide valid manipulation performance data.

• Normalization and median filtering: Normalized values within the (−1, 1) interval of the raw data were filtered in the median to eliminate interference noise from abnormal burrs. The filter used 30 points and a window length of 150 ms.
• Indexing: Each successive data portion that contains all values (above a threshold *T* or below −*T*) was indexed, resulting in no fewer than 14 valid manipulation data partitions, as instantiated by the red dots in Figure 1B. *T* defaulted to 0.8 and was manually adjusted in a few cases to obtain the correct number of manipulation repetitions.
• Averaging and deviation: The position deviation (PD) is the deviation between the mean value of all actual positions on all trails, as instantiated by the green baselines in Figure 1B and the theoretical value of the black circle, indicating the precision of the position. The repeatability of the position (PR) represents the standard deviation of all actual positions. The extreme range of the position (PE) means the range of operation on each side.

### 3.2 Preprocessing of sEMG data

Before extracting muscle synergies, the acquired sEMG signal was pre-processed.

• A mean shift procedure eliminated baseline shifts caused by trial or subject electrode displacements.
• A Butterworth filter of 20–250 Hz removed high-frequency noise, with which motion artifacts were also significantly eliminated.
• Fixed band-stop filters removed the frequencies of 50 Hz and 150 Hz.
• A full-wave rectification flipped the negative EMG signal above the baseline to ensure positive values.
• A Butterworth filter of 20 Hz filtered the rectified signal to provide a smooth envelope (Kieliba et al., 2018).
• Min-Max scaling normalized the signal.

The simultaneous use of sEMG and ACC signals is a paradigm often adopted for human activity studies (Liu and Schultz, 2022; Liu. et al., 2023). A single extraction have been performed over the whole trips (Oliveira et al., 2014). The acceleration signal (ACC) was interpolated due to different sampling rates and filtered from median to segment the results of muscle synergy activation. Pre-processed signals *V*_*m*×*t*_ (*m* is the channel of muscles) are decomposed:

$$V_{m\times t}≅W_{m\times n}\times H_{n\times t},$$ (1)

where *W* is the basis matrix for muscle activation, *n* is the number of muscle synergies, and *H* holds the coefficients of the activation of the *n* muscle synergies. *W*_*i*_ (*i* = 1, 2, …, *n*) is a vector of the muscle synergy matrix, each element of which takes a value between 0 and 1. *W*_*i*_s, specifying the participation level of each muscle in each synergy and forming a muscle synergy, are activated by the activation coefficient matrix *H*_*i*_, which represents the neural command of the CNS and determines the relative contribution of the muscle synergy matrix (Tang et al., 2017).

The reconstructed matrix $V_{m\times t}^{\prime }$ is assumed to be

$$V_{m\times t}^{\prime }=W_{m\times n}\times H_{n\times t},$$ (2)

which is directly related to the value of *n*. Variability accounted for (VAF) is defined as

$$\text{VAF}=1-\frac{{\left(V_{m\times t}-V_{m\times t}^{\prime }\right)}^{2}}{V_{m\times t}^{2}}$$ (3)

which could be used to decide the value of *n*. VAF quantifies the percentage of muscle cooperativity extracted relative to the original EMG signal. The literature shows that the signal is considered successfully reconstructed when the VAF is greater than 90% or until the addition of synergy does not further increase the VAF by an amount greater than 5% (Clark et al., 2010). The number of muscle synergies *n* ∈ [1, 10] is selected as the minimum number that could adequately reconstruct pre-processed signals in all trials (Tang et al., 2017).

### 3.3 Quantitative muscle synergy similarity

#### 3.3.1 Muscle synergy spaces

SCI (synergy coordination index) is used to evaluate the size of the resulting synergy space, in other words, the coordination between the synergies utilized. In class *c* ∈ {Experts, Trainees, Newbies}, the subject *k*’s synergy matrix *M*(*c*, *k*) and *H* (*c*, *k*) are extracted from the sEMG signals of all the corresponding trials. Each segmented ACC signal for muscle synergy activation is regarded as a circle, interpolated to 100 data points based on time.

$$M\left(c,k\right)=\left[\begin{array}{c} W_{1}\left(c,k\right)\\ W_{2}\left(c,k\right)\\ \vdots \\ W_{n}\left(c,k\right). \end{array}\right]$$ (4)

$$H\left(c,k\right)=\left[\begin{array}{c} H_{1}\left(c,k\right)\\ H_{2}\left(c,k\right)\\ \vdots \\ H_{n}\left(c,k\right) \end{array}\right]=\left[\begin{array}{cccc} \left[\begin{array}{c} H_{1_{m}}\left(c,k\right)\\ H_{2_{m}}\left(c,k\right)\\ \vdots \\ H_{n_{m}}\left(c,k\right) \end{array}\right] & \left[\begin{array}{c} H_{1_{m}}\left(c,k\right)\\ H_{2_{m}}\left(c,k\right)\\ \vdots \\ H_{n_{m}}\left(c,k\right) \end{array}\right] & \cdots & \left[\begin{array}{c} H_{1_{m}}\left(c,k\right)\\ H_{2_{m}}\left(c,k\right)\\ \vdots \\ H_{n_{m}}\left(c,k\right) \end{array}\right] \end{array}\right].$$ (5)

Only positive vector components have a cooperative space before the non-negative matrix decomposition for the estimation of *W*, Furthermore, the vectors *W*_*i*_ (*i* = 1, 2, …, *n*) are generally not orthogonal to each other. The size of the cooperative space depends on the relative angle of the vector *W*_*i*_. To quantify the size of the collaborative space, we define SCI in terms of the inner product of *W*_*i*_:

$$\text{SCI}\left(c,k\right)=\frac{2}{n\left(n-1\right)}\underset{i\neq j}{\sum }W_{i}\left(c,k\right)\cdot W_{j}\left(c,k\right).$$ (6)

SCI values range from 0 to 1. An SCI of 1 means that all vectors *W*_*i*_ are identical, while 0 means that all vectors *W*_*i*_ are orthogonal. The larger the SCI, the smaller the synergy space. The median and standard deviation values of SCI describe the synergy space of the group.

#### 3.3.2 Variability of muscle activation

In addition to computing the variability of *W*, an evaluation was also performed on the relationship between the variability of performance and the variability of synergy recruitment through the analysis of *H*, with the variability of the center of activity (CoA) in the form of standard deviation. The CoA of class *c* is defined as

$$\text{CoA}\left(c\right)=\left[\begin{array}{cccc} \text{CoA}\left(c,1\right) & \text{CoA}\left(c,2\right) & \cdots & \text{CoA}\left(c,K_{c}\right) \end{array}\right],$$ (7)

where *K*_*c*_ denotes the number of subjects in class *c* (*k* = 1, 2, …, *K*_*c*_) and

$$\text{CoA}\left(c,k\right)=\left[\begin{array}{c} {\text{CoA}}_{H_{1}}\left(c,k\right)\\ {\text{CoA}}_{H_{2}}\left(c,k\right)\\ \vdots \\ {\text{CoA}}_{H_{n}}\left(c,k\right) \end{array}\right].$$ (8)

${\text{CoA}}_{H_{i}}\left(c,k\right)$ is calculated as the mean of 14 cycles cut from the ACC signal:

$${\text{CoA}}_{H_{i}}\left(c,k\right)=\frac{1}{14}\overset{14}{\underset{t=1}{\sum }}\left({\text{CoA}}_{H_{i_{m}}}\left(c,k\right)\right),$$ (9)

where the CoA during the *m*th part of *H*_*i*_(*c*, *k*), ${\text{CoA}}_{H_{i_{m}}}\left(c,k\right)$ , was calculated using circular statistics (Watson, 1982; Kibushi et al., 2018a) and plotted in polar coordinates with polar directions of 0°–360° and radii denoting the mean activities of the muscle:

$${\text{CoA}}_{H_{i_{m}}}\left(c,k\right)=\tan^{-1}\frac{\sum_{t=1}^{100}\left.\left(\sin\theta \times H_{i_{m}}\left(t\right)\right)\right)}{\sum_{t=1}^{100}\left.\left(\cos\theta \times H_{i_{m}}\left(t\right)\right)\right)}.$$ (10)

${\text{CoA}}_{H_{i_{m}}}\left(c,k\right)$ was calculated as the vector angle, the first trigonometric moment that points to the center of mass of that circular distribution using the following formulas. Variability is reflected in the standard deviation (STD):

$${\text{STD}}_{H_{i_{m}}}\left(c,k\right)=\sqrt{\frac{1}{13}\overset{14}{\underset{i=1}{\sum }}{\left({\text{CoA}}_{H_{i}}\left(c,k\right)-\bar{{\text{CoA}}_{H_{i}}\left(c,k\right)}\right)}^{2}}.$$ (11)

### 3.4 Statistical analysis

The descriptive statistics in this work involve mean values and standard deviations. Four one-way repeated analyses of variance (ANOVA) were performed on the synergy similarity and manipulation performance metrics (PR, PD, PE) among the three classes. In addition, the Pearson correlation coefficient *r* was analyzed through the manipulation performance (PR_left_, PR_right_, PD_left_, PD_right_, PE_left_, and PE_right_), SCI, ${\text{CoA}}_{H_{i_{m}}}$ , and ${\text{STD}}_{H_{i_{m}}}$ . The significance level was set as *p* < 0.05 for the statistical analysis implemented by *SPSS* (version 26.0, SPSS Inc. Chicago, IL, United States).

## 4 Results

### 4.1 Statistical analysis of manipulation performance metrics

**FIGURE 3:**
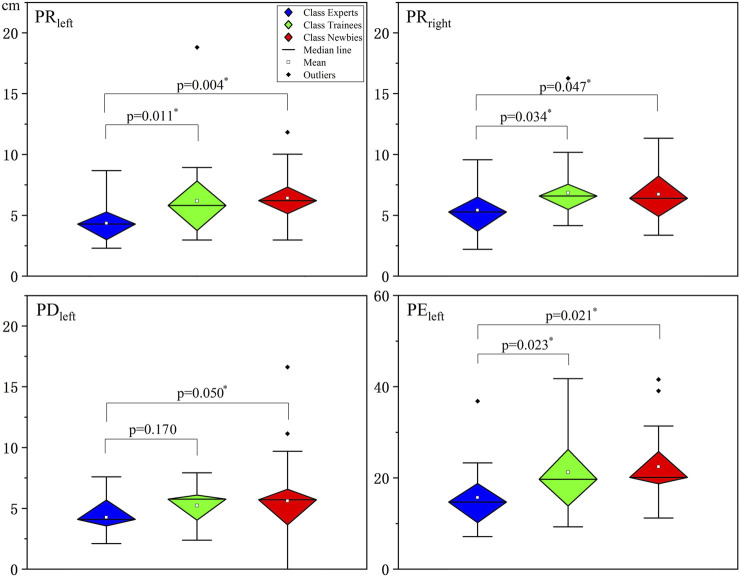
Significant results of the three classes’ manipulation performance metrics analyzed by one-way analyses of variance (ANOVA). The vertical axes indicate the error value. All data passed the homogeneity of variance test. The asterisks indicate high significance (*p* < 0.05). PR: repeatability of the position; PD: position deviation; PE: extreme range of the position. Note that PE_left_ differs from the other three subplots in the vertical axis range.

The performance metrics for the manipulation of the three classes, PR, PD, and PE, are analyzed by one-way ANOVA in Figure 3, where all data passed the homogeneity of variance test. Significant differences were discovered in the values of PR_left_ (*F* = 4.955; *p* = 0.010), PR_right_ (*F* = 2.735; *p* = 0.071), and PE_left_ (*F* = 3.415; *p* = 0.038) between the three classes. Note that the asterisk indicates high significance (*p* < 0.050), as does the following. Comparing each pair of classes yields the following findings.

PR_left_ and PR_right_

Compared to Experts, Trainees (Δ = −1.80 ± 0.70; *p* = 0.011) and Newbies (Δ = −2.07 ± 0.70; *p* = 0.004) have larger PR-left values; However, Figure 3(PR_left_) shows no significant difference between Trainees and Newbies. The case of PR_right_ is similar: Trainees (Δ = −1.43 ± 0.66; *p* = 0.034) and Newbies (Δ = −1.32 ± 0.62; *p* = 0.047) are not significantly different, while both are larger than Experts, as diagnosed in Figure 3(PR_right_).

PD_left_

Newbies’ PD_left_ (Δ = −1.35 ± 0.70; *p* = 0.050) is significantly larger than Experts’ PD_left_. At the same time, Trainees’ PD_left_ did not show a significant difference (Δ = −0.96 ± 0.70; *p* = 0.177) compared to Newbies’ and Experts’ results, as reflected in Figure 3(PD_left_).

PE_left_

The relationship of the PE_left_ difference between Experts, Trainees (Δ = −6.95 ± 2.90; *p* = 0.021), and Newbies (Δ = −6.70 ± 2.90; *p* = 0.023) is the same as in the case of PR_left/right_, which can be verified by Figure 3(PE_left_).

### 4.2 Muscle synergy extraction

**FIGURE 4:**
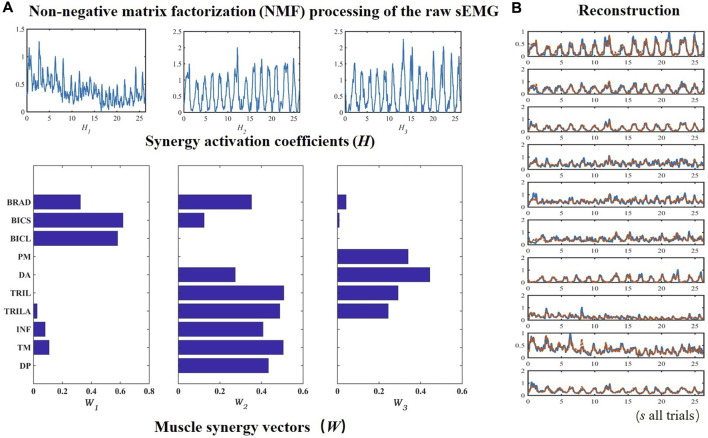
An example of extracting muscle synergies on ten upper limb muscles from Trainees. The sEMG data from ten upper limb muscles were processed by non-negative matrix factorization **(A)**, based on which synergy activation coefficients and muscle synergy vectors were used to reconstruct the sEMG **(B)**.

Figure 4 instantiates the non-negative matrix factorization (NMF) decomposition process of ten upper limb muscles. The basis matrix of muscle activation *W* and the activation coefficient matrix *H* are shown in Figure 4A.

**FIGURE 5:**
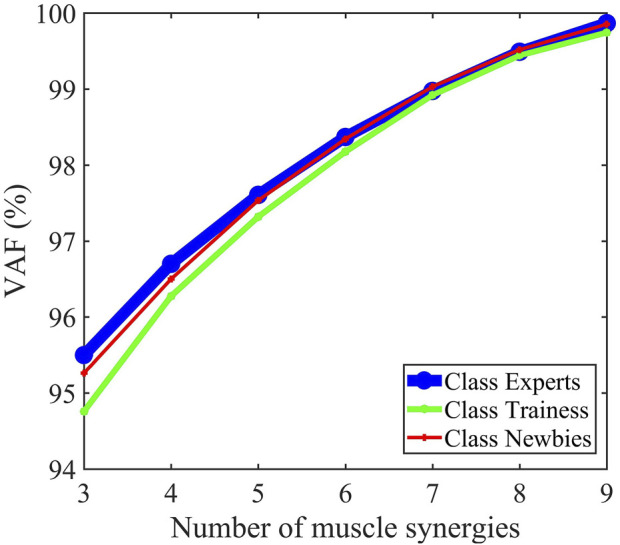
Mean percentage of variability accounted for (VAF) as a function describing the number of extracted synergies. Three synergies were identified for the three classes.

Figure 4B evinces that the synergy *W*_1_ between the muscle synergy vectors *W* mainly reflects the activation of BRAD, BICS, and BICL, whose synergies are greater than 0.3. *W*_2_ primarily comprises DP, TM, INF, TRIL, TRILA, BICL, and BRAD. The dominant composition elements of *W*_3_’s are DA, PM, TRILA, and TRIL. The sEMG could be reconstructed by *H* and *W*, as manifested with the raw sEMG of ten muscles of the upper limbs in Figure 4B. Figure 5 revealed that after the number of synergies reaches three, the mean VAF of each class exceeds 95%.

### 4.3 Intraclass muscle synergy extratcion

**FIGURE 6:**
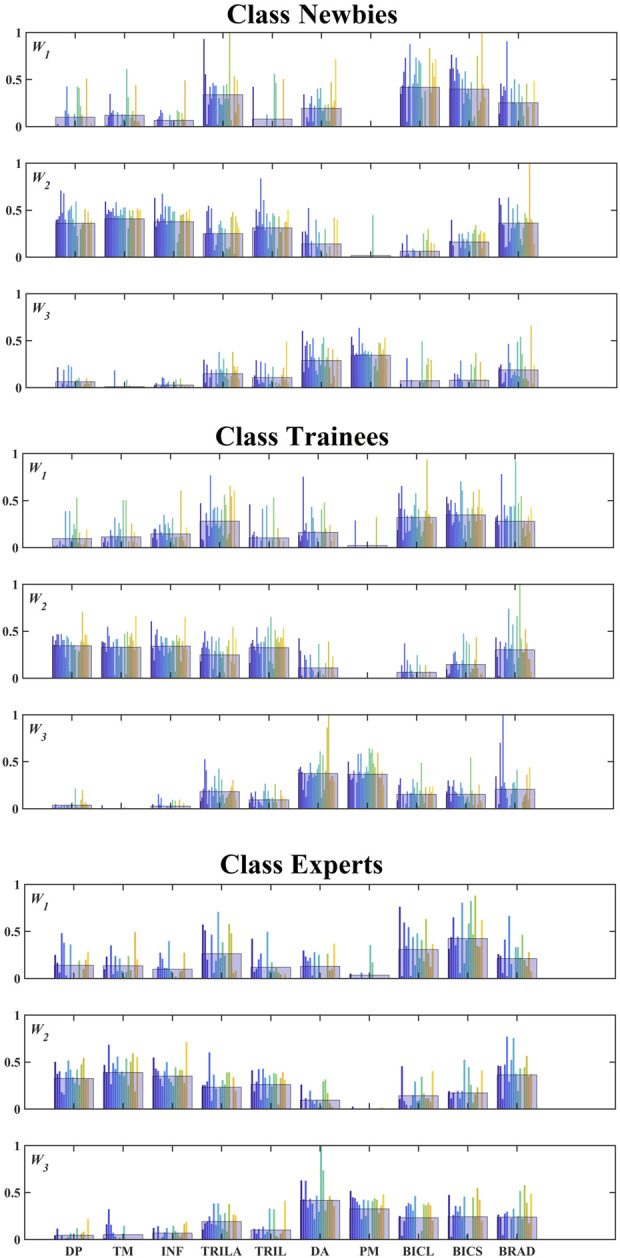
Composition of muscle synergies for the three classes. Three synergies were extracted from all subjects and matched among the classes. The colorized bars indicate different subjects. Note that the numbers of subjects in the three classes are 19 (Experts), 30 (Trainees), and 28 (Newbies). The black-bordered wide bar for each muscle indicates the mean value.

**FIGURE 7:**
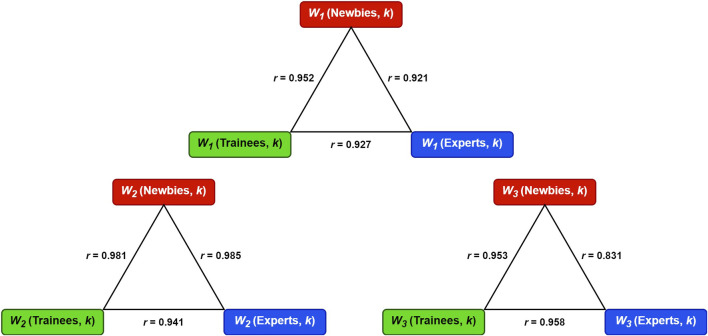
The Pearson correlation coefficients (*r*) among the three classes for synergies *W*_1_, *W*_2_, and *W*_3_.

The colorized bars in Figure 6 indicate different subjects in classes Experts (19 subjects), Trainees (30 subjects), and Newbies (28 subjects), and the black bars represent the mean values among each class for each muscle. PM has a low proportion in both INF *W*_1_ and *W*_2_, while DP, TM, INF, and TRIL exhibit low values in *W*_3_, found in all three classes. Figure 7 embodies the Pearson correlation coefficients among the three classes for synergies *W*_1_, *W*_2_, and *W*_3_. Figures 6, 7 show that the three classes have the same muscle synergies patterns, occurring in the same order.

### 4.4 Muscle synergy space

**FIGURE 8:**
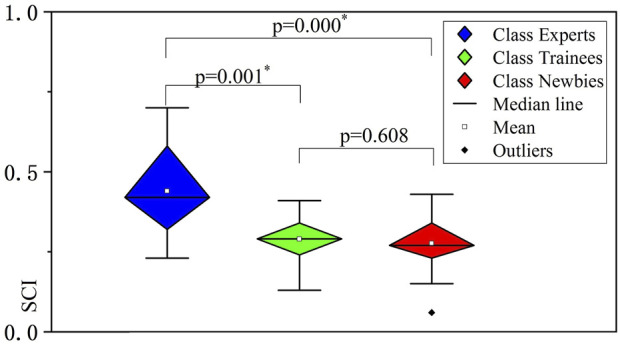
Significance results of the three classes’ spaces of muscle synergies analyzed by nonparametric test. The vertical axes indicate the synergy coordination index (SCI) values. The asterisks indicate high significance (*p* < 0.05).

In addition to the three extracted muscle synergies elucidated in Figure 6, Figure 8 presents the SCI values of the three classes, where SCI(Experts) is 0.419 ± 0.138, SCI(Trainees) is 0.290 ± 0.070, and SCI(Newbies) is 0.270 ± 0.087, respectively. The SCI value of experts was significantly higher (*p* = 0.000, *p* = 0.001) than the values of trainees and newbies, between which no significant difference (*p* = 0.608) can be concluded.

### 4.5 The center of synergistic activities

**FIGURE 9:**
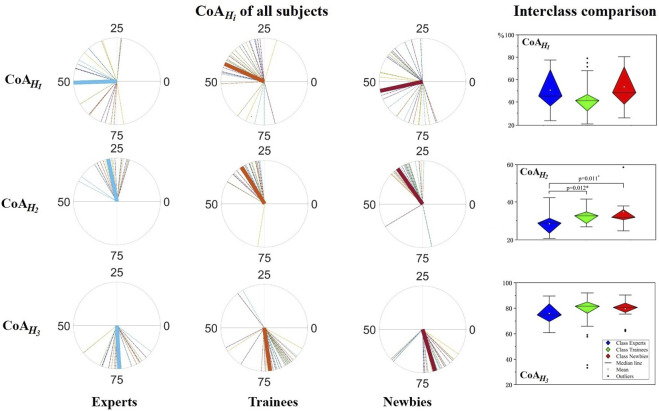
Center of activity (CoA) analysis for muscle synergy. Left: ${\text{CoA}}_{H_{i}}$ among subjects across the whole experiment, where the averaged ${\text{CoA}}_{H_{i}}$ values of subjects lie in the polar coordinate, and the polar directions denote the relative time over each trial cycle with clockwise time progression; Right: significance results of the three classes’ ${\text{CoA}}_{H_{i}}$ s analyzed by nonparametric test, where the vertical axes indicate ${\text{CoA}}_{H_{i}}$ values. The asterisks indicate high significance (*p* <0.05).

Figure 9 exhibits the resulting CoA values and the interclass comparison. ${\text{CoA}}_{H_{1}}$ , related to the flexion and extension of the elbow, shows no significant differences between the three classes. A similar case is ${\text{CoA}}_{H_{3}}$ , recruited during the pronation of the forearm and shoulder. It should be noted that for ${\text{CoA}}_{H_{2}}$ , recruited during supination of the forearm and shoulder, the activation of experts changed to an earlier phase (*p* = 0.012, *p* = 0.011).

**FIGURE 10:**
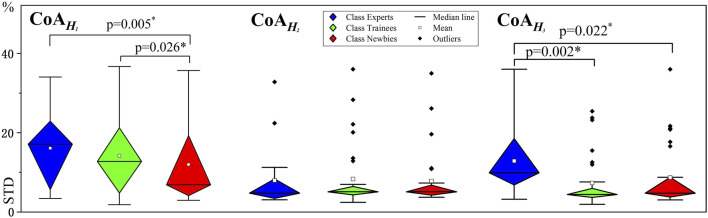
Variability analysis for muscle synergy. The vertical axes indicate standard deviation (STD) values. The asterisks indicate high significance (*p* < 0.05).

Figure 10 provides a variability comparison between the three classes by STD values. Experts’ intraclass variability of ${\text{CoA}}_{H_{3}}$ , that is, standard deviation, is more pronounced than Trainees’ (*p* = 0.022) and Newbies’ (*p* = 0.002), while there is no significant difference between the latter two. As a point of comparison, in ${\text{CoA}}_{H_{1}}$ , Experts’ STD is more evident than Newbies’ (*p* = 0.005), while an insignificant difference is shown between Experts and Trainees.

### 4.6 Correlation between manipulation performance and analytical metrics

**FIGURE 11:**
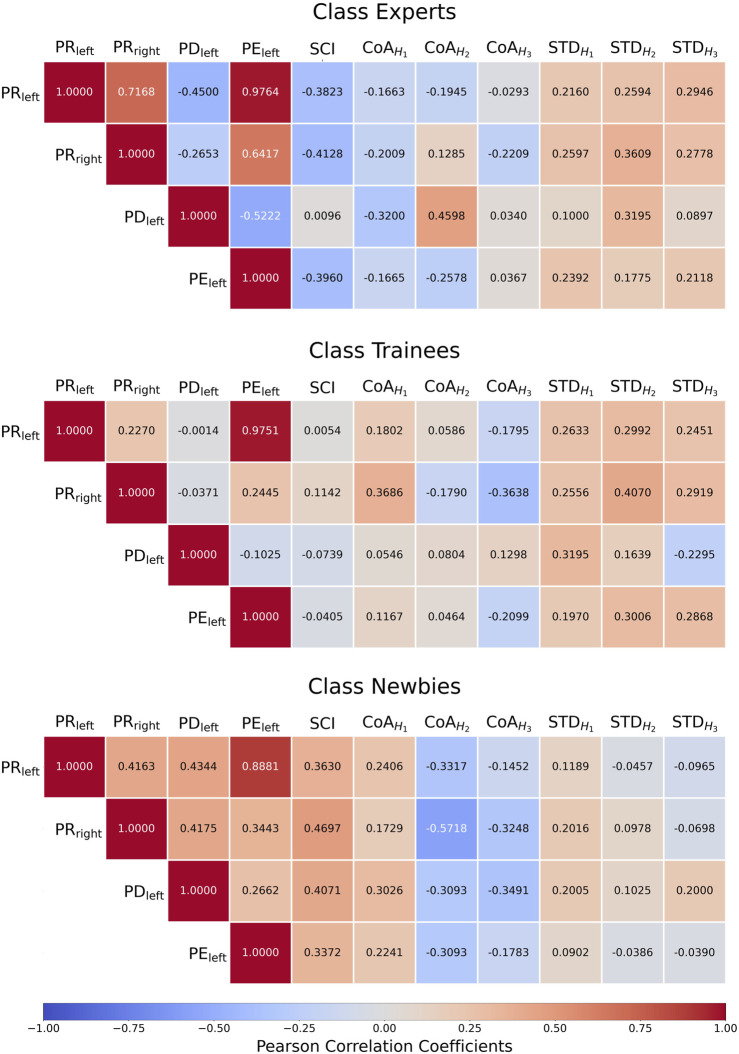
Three classes’ heatmaps of the Pearson correlation coefficients between manipulation performance metrics and synergy coordination index (SCI)/the center of activity (CoA)/CoA’s standard deviation (STD).

Figure 11 details the Pearson correlation coefficients between manipulation performance metric and SCI/CoA/STD. In general, the results of Experts often show significant negative correlation values between the manipulation performance metrics PR_left_/PR_right_/PE_left_/PE_right_ and SCI/CoA.

#### 4.6.1 Manipulation performance *versus* SCI

For Experts, PR_left_, PR_right_, and PE_Left_ in manipulation performance metrics show high negative correlations with SCI (−0.3823, −0.4128, and −0.3960), and PD_left_ has a positive, close to zero correlation with SCI (0.0096). On the contrary, newbies collectively have high positive correlations between PR_left_/PR_right_/PD_left_/PE_Left_ and SCI (0.3630, 0.4697, 0.4071, and 0.3397). The absolute correlations of the trainees are low (−0.005, −0.1142, −0.0739, −0.0405). The experimental results are consistent with the important muscle learning methodology (Alnajjar et al., 2013). Newbies’ muscle status *W*) shows variability, which SCI reflects, whereas Experts tend to stabilize.

#### 4.6.2 Manipulation performance *versus* CoA

Newbies show negative correlations between the ${\text{CoA}}_{H_{2}}$ / ${\text{CoA}}_{H_{3}}$ values and the four manipulation performances, of which the *r* values fell between −0.5718 and −0.1452, while the correlations between ${\text{CoA}}_{H_{1}}$ and the four manipulation performances are positive. Trainees’ manipulation- ${\text{CoA}}_{H_{1}}$ correlations are significantly lower than Newbies, except for PR_right_, though still all positive. Experts’ manipulation- ${\text{CoA}}_{H_{1}}$ correlations go the opposite, all negative (*r* = −0.3200–−0.1663). No general pattern was found in the remaining comparisons.

#### 4.6.3 Manipulation performance *versus* STD (standard deviation of CoA)

The manipulation performance of Experts is positively correlated with the standard deviation of CoA in *H*_1_–*H*_3_ (*r* = 0.0897–0.3609). Trainees’ STD values are essentially similar to Experts’, except that the PD_left_- ${\text{STD}}_{H_{3}}$ correlation is negative. For Newbies, the correlations are a mixture of positive and negative values, generally lower than Experts’ and Trainees’ values, except for two PD_left_-related correlations.

## 5 Discussion

Variability is a natural component of human movement. The notion of muscle synergy variability contains a lower-dimensional synergy space and time-dependent variables. Oriented towards better operation, variability affects the performance of motor tasks. As muscle synergy spaces are gradually optimized, movement performance will improve.

Based on the muscle synergy analysis of joystick manipulation in three classes of maneuvering experience, this study reveals the correlation between manipulation performance and muscle synergy variability, reflected by SCI and the standard deviation of CoA.

### 5.1 Structure of muscle synergies

The synergy structure corresponds to the synergy number, of which the patterns are related to the task. Consistency is demonstrated in different individuals. In this study, three muscle synergies were investigated, consistent with previous studies. Three to five muscles work together in the three-dimensional force generation of the upper extremity (Coscia et al., 2014). Four to five synergies can reconstruct the activation patterns of up to 19 muscles recorded during point-to-point reaching movements or forearm postures with different loads (d’Avella et al., 2003). In comparison, three synergies are sufficient to reconstruct multijoint movements performed in the horizontal plane (Roh et al., 2012). The consistency of the synergy number could be affected by individual differences such as muscle size, strength, subcutaneous fat thickness, and measurement electrode position, which is also in line with previous work. In addition to the physiological factors mentioned above, the synergy number may also be influenced by sEMG pre-processing. The variance accounted for (VAF) criterion is sensitive to the low-pass cut-off frequency, with higher cut-offs resulting in lower VAF (Kieliba et al., 2018).

The pattern of muscle synergies studied in this work is qualitatively similar to those underlying three-dimensional force generation (Roh et al., 2012). Their flexion pattern consisting of BRAD and BI with DP activity is similar to *W*_1_ identified in our experiments, while their flexion pattern comprised of TRILA, TRIL, and PM is similar to the *W*_3_ (see Figure 4). Figure 6 exhibits that neuromuscular strategies remain the same in the three classes, interpreting that joystick manipulation experience does not cause variations in the pattern of muscle synergies. Previous studies on lifting or shoulder movement had similar results: Muscle synergy is consistent between experts and normal people during lifting (Zhao et al., 2021) or shoulder movement (Turpin et al., 2020). In general, the structure of the muscle synergy was influenced by biomechanics and task constraints (Roh et al., 2012).

### 5.2 Muscle synergy variability

Muscle synergy spaces vary with training. Previous studies have suggested that muscle synergies may be formed by adaptive processes related to the individual’s experience. The activity of the primary motor cortex is adapted with training, associated with changes in either the amplitude of activation or the composition of the correlated synergy. As a result of maintaining balance, the body will achieve a stable pattern of muscle synergy and variable activation after training (Alnajjar et al., 2013). CNS searches for the most appropriate synergy space region, evoking various strategies by tuning *W*. In such a search stage, low stability of *W* is observed. The modulation of the muscle synergy composition will develop to a stable state during skill learning (Kargo and Nitz, 2003) (see Figure 8). Experts showed higher SCI, representing smaller muscle synergy spaces and more excellent stability. The relatively concentrated muscle synergy spaces observed among experts should be explained by their long-term maneuvering experience, which enables their CNS to create optimal sets of efficient behaviors by optimizing the size of the synergy space in the appropriate region (Alnajjar et al., 2013).

In addition to space properties, the variability of temporal activation is also affected by training. Previous studies have shown that CoA changes with walking speed (Kibushi et al., 2018b), load (Labini et al., 2011; Sylos-Labini et al., 2014), and cerebellar ataxia (Martino et al., 2014), among others. This study reveals a significant shift in ${\text{CoA}}_{H_{2}}$ between Experts and other subjects (see Figure 9). Literature showed that during the bench press, experts’ variability of the coactivation coefficient is higher than normal subjects’, while the muscle coactivation vector shows low variability (Kristiansen et al., 2015). In our study, Experts showed higher variability (STD) in ${\text{CoA}}_{H_{3}}$ , which contributed to the pronation of the forearm and shoulder to acquire better position accuracy of the left position (see Figure 10): Moreover, Experts’ variability (STD) in ${\text{CoA}}_{H_{1}}$ is also more obvious. After learning, *W* becomes more stable in a particular region and smaller, and the constraint on the variability of *H* gradually eases (Alnajjar et al., 2013).

### 5.3 Significant negative correlation between manipulation performance metrics and muscle synergy variability

A smaller synergy space is often used for a better performance, which can answer why the coordinated movements respond to disturbance (Alnajjar and Shimoda, 2017). In this study, Figure 11 evinces that the stability of the muscle synergy space is positively correlated with the manipulating error in the novice stage (Newbies) and negatively correlated in the veteran stage (Experts). On Experts, the smaller the synergy space, the better the performance. In contrast, Newbies are in the search stage of *W*, so the larger the synergy space, the better the performance.

The CNS learning model suggests that the CNS attempts to reduce the degrees of freedom of the resulting motions by restricting the variability of the synergy coefficient matrix *H*) during the search stage of *W* and simplifies the handling of its high temporal variability (Alnajjar et al., 2013). Therefore, Newbies’ correlation between CoA and manipulation performance is more significant than that of the other two classes. In the Experts and Trainees classes, no significant correlation is detected between CoA and manipulation performance. Consistent with previous studies, manipulation performance can reflect proficiency degree: Once a certain proficiency level is reached, the body’s restrictions on muscle activation will be lifted, endowing a complete utilization of the body’s potency (i.e., *W* variability decreases, *H* variability increases) (Alnajjar et al., 2013).

Expert’s and Trainees’ variability (STD) of ${\text{CoA}}_{H_{1}}$ and ${\text{CoA}}_{H_{3}}$ is more pronounced than Newbies’ (see Figure 10). Although muscle activation variability decreased with increasing training degrees interclass-wise, a positive correlation can be found between variability and manipulation performance intraclass-wise. The intraclass analysis discloses that correlation values increase as experience improves and that the significance levels of the three classes are low. Research has ascertained that many compensatory solutions exist for different motor tasks and that various solutions can generate the same movement (Ting and Macpherson, 2005; McKay and Ting, 2008). The CNS adjusts motor impedance by activating antagonistic muscles to minimize the interference effect caused by load, thus improving movement accuracy (Burdet et al., 2001; Franklin et al., 2007).

## 6 Conclusion

This article analyses manipulation performance and muscle synergy in three classes of subjects with different maneuvering experiences. Different levels of experience and training lead to apparent differences in the space size of *W* and the variability of *H*. The precision of the manipulation is related to the variability of muscle synergy. Long-term training compresses muscle synergy space, which enhances muscle manipulation performance. Experts have significantly higher muscle synergy space stability and activation variability than the other two classes. Experts’ and Trainees’ correlation values between manipulation performance and the standard deviation of CoA are more significant than Newbies’.

This work has designed experiments, grouped 77 subjects, collected multimodal data, and conducted various experimental analyses, all of which have informed subsequent research. Our findings and validations are looked to for progressive practical applications that can provide important references for the development of software to aid pilot training, such as tips and training procedures to reduce manipulation errors as well as training result analysis and evaluation, which can improve efficiency, save costs, and potentially increase the safety of flight.

## Data Availability

The raw data supporting the conclusion of this article will be made available by the authors upon request, without undue reservation, except for commercial use.
